# Fast vortex dynamics and relaxation times in NbRe-based heterostructures

**DOI:** 10.3762/bjnano.17.20

**Published:** 2026-02-12

**Authors:** Francesco De Chiara, Zahra Makhdoumi Kakhaki, Francesco Avitabile, Francesco Colangelo, Abhishek Kumar, Carmine Attanasio, Carla Cirillo

**Affiliations:** 1 Dipartimento di Fisica “E. R. Caianiello”, Università degli Studi di Salerno, I-84084 Fisciano (Sa), Italy; 2 Institute for Electrical Measurement Science and Fundamental Electrical Engineering, Technische Universität Braunschweig, D-38106 Braunschweig, Germany; 3 CNR-SPIN, c/o Università degli Studi di Salerno, I-84084 Fisciano (Sa), Italy; 4 Centro NANO_MATES, c/o Università degli Studi di Salerno, I-84084 Fisciano, Salerno, Italy

**Keywords:** flux-flow instability, quasiparticle relaxation time, superconducting heterostructures, superconductivity, vortex dynamics

## Abstract

An in-depth analysis of Abrikosov vortex dynamics and flux-flow instabilities was performed in NbRe/Au and NbRe/Py bilayers to compare superconducting/normal metal (S/N) and superconducting/ferromagnetic (S/F) heterostructures based on the same superconducting layer. The heterostructures, fabricated by sputtering, were characterized through electrical transport measurements. The *I*–*V* characteristics show that, in the NbRe/Py bilayer, vortices reach higher critical velocities than those observed in the NbRe/Au structure. The analysis of the flux-flow instability within the Larkin–Ovchinnikov framework allows one to extract the quasiparticle energy relaxation time. For external magnetic field values for which edge barrier pinning is dominant and thermal effects are negligible, the relaxation times are about 150 ps and 24 ps for NbRe/Au and NbRe/Py bilayers, respectively. These results indicate that NbRe/Py bilayers, having a relaxation time one order of magnitude smaller than values reported in NbRe microbridges, have great potential for the realization of devices where fast relaxation processes are required.

## Introduction

Vortex dynamics plays a central role in the electrical transport under magnetic fields in type-II superconductors and is an essential subject of research in superconductivity [1]. In inhomogeneous type-II superconductors, structural defects create pinning centers that trap vortices. As a result, a finite current density is required to initiate vortex motion. When the value of the external current density, *j*_ex_, applied to the superconductor exceeds the value of the critical current density, *j*_c_, vortices begin to move, giving rise to a viscous flow regime known as flux flow, which is associated with energy dissipation [2]. As the current increases further, *j*_ex_ ≫ *j*_c_, and as the vortex velocity approaches a maximum critical value, this regime may become unstable. A sudden voltage jump is observed in the *I*–*V* characteristic, attributed to the collapse of superconducting coherence. This phenomenon, known as flux-flow instability (FFI), depends on several factors such as structural disorder and defects [3], pinning mechanisms [4–5], thermal effects [6], and sample geometry [7]. FFI is effectively described by the Larkin–Ovchinnikov (LO) model [8], which relates the onset of the instability to the maximum value of vortex velocity, v*, and the quasiparticle relaxation time, τ_E_. The latter characterizes how efficiently quasiparticles (unpaired electrons) return to their equilibrium state from a non-equilibrium one [9]. τ_E_ represents a key parameter for the optimization of superconducting quantum technologies, including superconducting detectors [10–12], superconducting qubits [13–14], and superconducting nano- and microstrips used in high-speed electronics [15], as well as for the development of hybrid architectures enabling magnon–fluxon interactions [16]. The enhancement of vortex dynamics and the reduction of τ_E_ are strongly linked to the optimization of quasiparticle relaxation mechanisms. Excited quasiparticles can relax primarily through two processes, namely thermal electron–phonon (e–ph) interaction and electron–electron (e–e) recombination [1]. In classical, low-*T*_c_ superconductors, the dominant relaxation channel is provided by e–ph scattering events [17], while in high-*T*_c_ superconductors the situation is reversed, with e–e recombination playing a major role in energy relaxation [18]. The efficiency of these processes and the value of τ_E_ strongly depend on the microscopic properties of the superconductor and on the degree of electronic disorder [1]. In this context, NbRe has emerged as a promising material that exhibits exceptionally fast vortex dynamics [19]. Extensive structural characterizations performed by X-ray diffraction have shown that NbRe thin films are polycrystalline with oriented grains of nanometric size, of the order of a few nanometers [20–21]. Furthermore, earlier investigations have demonstrated that the superconducting and transport properties of NbRe are robust against moderate thickness variations [19]. These characteristics, together with disorder-dominated transport, favor very short temperature-independent quasiparticle relaxation times of the order of hundreds of picoseconds [22] making NbRe-based films particularly attractive for studying non-equilibrium effects and for the realization of fast superconducting devices [23–25].

To modify quasiparticles relaxation mechanism, one of the most promising strategies involves engineering hybrid heterostructures in which the superconducting layer (S) is brought into contact with a normal metal (N) or a ferromagnetic layer (F). Several studies have shown that, in S/N [26–30] or S/F [31–34] systems, different mechanisms can influence the vortex dynamics depending on the thickness and conductivity of the capping layer. In particular, a sufficiently thick metallic overlayer may lead to a damping of vortex motion due to eddy currents induced by the time-dependent magnetic flux, resulting in a magnetic breaking effect [29–30]. In the opposite limit of thin capping layers, the proximity effect plays a dominant role leading to an enhancement of the critical vortex velocity and promoting faster relaxation processes [26–28].

In this work, we systematically investigate vortex dynamics and flux-flow instability phenomena in micrometer-wide bilayers in which NbRe is brought into contact with either thin N or F materials. In particular, we measure transport properties in the presence of a magnetic field in NbRe/Au and NbRe/Py microstrips. Experimental data show that the two materials modify the vortex dynamics of NbRe differently, with Py allowing for larger v* as a function of the magnetic field. Furthermore, the calculation of the quasiparticle energy relaxation times reveals faster relaxation times in NbRe/Py than in NbRe/Au. This result demonstrates the larger effectiveness of ferromagnetic materials in further promoting the energy relaxation process.

## Larkin and Ovchinnikov Theory

As pointed out by Larkin and Ovchinnikov [8], FFI originates from the finite energy relaxation time of quasiparticles, which causes significant variations in the quasiparticle distribution function, *f*(*E*). During vortex motion, quasiparticles inside the vortex core are accelerated by the electric field associated with flux flow and undergo successive Andreev reflections at the core boundaries [35]. As their energy approaches the temperature-dependent superconducting gap, Δ(*T*), quasiparticles diffuse into the surrounding superconducting region, reducing the number of excitations within the core. This process results in a shrinkage of the vortex followed by a decrease of the viscous drag force, *f*_vd_, and an increase in the vortex velocity *v* [9]. When *v* exceeds the critical value v*, the Lorentz force dominates the viscous drag force, leading to instabilities in vortex motion. The dependence of v* on *T* is given by:


[1] $$v^{*}=\frac{D^{1/2}{\left[14\zeta \left(3\right)\right]}^{1/4}}{{\left(\pi \tau_{\text{E}}\right)}^{1/2}}{\left(1-\frac{T}{T_{\text{c}}}\right)}^{1/4},$$

where *T*_c_ is the superconducting critical temperature, *D* is the quasiparticles diffusion coefficient, and ζ(*x*) is the Riemann zeta function. The critical velocity is inversely proportional to the square root of the inelastic scattering time τ_E_, and it is independent on the external magnetic field. This result follows from the core assumption of the LO theory, namely that *f*(*E*) is spatially uniform within the superconductor. Consequently, while the vortex core contracts, its shape preserves axial symmetry. Furthermore, the flux-flow instability nucleates simultaneously throughout the superconductor, leading to a field-independent critical velocity v* at which the instability is established [1]. It is worth emphasizing that, since the nucleation of the flux-flow instability is strongly influenced by the quality of the sample edges, the estimation of an intrinsic quasiparticle relaxation time from Equation 1 is justified only when the field dependence of the critical current evidences a dominant edge-barrier pinning mechanism. In this regime, the assumptions underlying the LO model remain valid and the deduced relaxation time can be regarded as an intrinsic property of the superconducting system [1].

Experimentally, biasing a superconducting strip with a current *I*, leads, in the flux flow regime, to the appearance of a jump to normal state at an instability current I*, which corresponds to the instability voltage V*. I* is usually smaller than the depairing current, *I*_d_, and, therefore, sets the actual limit for the use of superconductors in applications [1]. When the Larkin–Ovchinnikov instability develops from a well-established flux-flow regime, characterized by a homogeneous vortex motion, the instability voltage allows one to quantify the critical vortex velocity using the relation v* = V*/(μ_0_*HL*) [5,9] and then, using Equation 1, to calculate the relaxation time τ_E_ and estimate the lifetimes of electronic excitations in superconductors.

### S/N and S/F bilayer

One way to enhance the critical vortex velocity and reduce the relaxation time is to use multilayer superconducting heterostructures [28,33]. It was experimentally observed that thin superconducting strips coated by a lowly resistive normal metal present stronger non-linear properties and higher vortex velocity than a single superconducting strip [27]. This result may be attributed to proximity-induced superconductivity in the normal layer. At the interface, superconductivity is induced into the normal metal over the coherence length


[2]

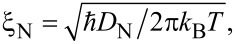



where *D*_N_ = *v*_F_*l*/3 [36] is the electron diffusion coefficient in the normal layer with *v*_F_ the Fermi velocity and *l* the electron mean free path. For typical Fermi velocities [36] and low-temperature mean free paths measured in normal metals, ξ_N_ lies in the range of tens to hundreds of nanometers. According to Belzig et al. [37], the superconducting correlation in N produces a minigap at the Fermi energy, smaller than the superconducting gap of S and related to the thickness of the N layer, *d*_N_, by the relation


[3]

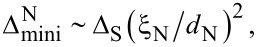



where Δ_S_ is the superconducting gap of the S layer. Since in the S/N bilayers ξ_N_ ≤ *d*_N_, quasiparticles will be more easily removed from the vortex core, and due to their lower energy, they will relax faster. This implies smaller τ_E_ and higher critical velocity.

Based on these considerations, it is expected that coupling the superconductor with a ferromagnetic material should lead to a further increase in critical velocity, as experimentally observed [31–34]. In S/F bilayers, although no minigap is formed in the ferromagnetic layer, superconducting correlations can penetrate into F, but over much shorter distances compared to a normal metal. In the dirty limit, the characteristic coherence length ξ_F_ can be written as [38]


[4]

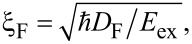



where *D*_F_ and *E*_ex_ are the electron diffusion coefficient and the ferromagnet exchange energy, respectively. Since, at low temperatures, *E*_ex_ (≈0.01–1 eV) largely exceeds the thermal energy, *k*_B_*T*, ξ_F_ is typically of the order of 1–5 nm [33]. As a result, in contrast to the much longer decay observed in S/N systems, superconducting properties in S/F bilayers are confined to just a few nanometers in the ferromagnetic layer where the overall superconducting properties are very much suppressed. Consequently, S/F hybrids are expected to exhibit reduced quasiparticle relaxation times and enhanced critical velocities compared to S/N bilayers.

Finally, in addition to the spectral argument, general considerations related to increased scattering rates should also be considered. In S/N and S/F systems, additional scattering channels become available for quasiparticles both in the proximized superconducting layer and in the adjacent region where superconducting correlations are induced. In particular, Ryan and Chandrasekhar [26] have shown that a thin disordered normal layer enhances the inelastic scattering rate, leading to a significant reduction of the effective relaxation time without degrading superconductivity. This finding supports the idea that both spectral and kinetic proximity effects cooperate in promoting faster quasiparticle relaxation in hybrid systems.

## Experimental

Bilayers were deposited by DC magnetron sputtering in an ultrahigh vacuum system at room temperature from Nb_0.18_Re_0.82_ (NbRe), Au, and Ni_0.80_Fe_0.20_ (permalloy, Py) targets on SiO_2_ substrates. The base pressure was *P* = 2.2 × 10^−8^ mbar, and the Ar pressure during the deposition was 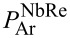 = 6.6 μbar, 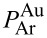 = 12 μbar and 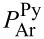 = 6.3 μbar. During the deposition, the film thickness was monitored in situ by a thickness monitor, which provides a real-time measurement of the growth rate. The deposition rates were later calibrated ex situ using a Bruker Dektak XT stylus profilometer by measuring step heights in samples. In the NbRe/Au sample, the NbRe layer has a thickness 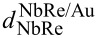 = 20 nm, while 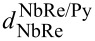 = 15 nm in the NbRe/Py sample. The corresponding Au and Py layers are *d*_Au_ = 5 nm and *d*_Py_ = 4 nm. Given the ultrathin thickness of the metallic overlayers, electromagnetic damping effects due to eddy currents are expected to be strongly reduced, placing the present samples in the thin-layer regime where proximity-induced effects are dominant [29–30]. The samples were patterned into a four-probe geometry by optical lithography using a direct laser writer exposure followed by an ion-etching procedure. This technique results in microstrips with straight edges, ensuring a uniform width, *w*, of the device [39]. The geometrical values of the microstrips are reported in Table 1. Electrical contacts were realized using an ultrasonic wire bonder, ensuring a highly reproducible and comparable contact quality for both NbRe/Au and NbRe/Py microbridges. The electrical transport measurements were performed using a cryogen-free measurement system from Cryogenic Ltd. The samples were mounted in the measurement system with the surface perpendicular to the applied field. Resistive transition measurements were performed using a DC bias current *I*_b_ = 10 μA. For all samples, the critical temperature was defined as the temperature at which the resistance drops to 50% of the normal-state value measured at *T* = 10 K (*R*^10K^). Finally, *I*–*V* characteristics were measured as a function of the applied magnetic field at the reduced temperature *t* = *T*/*T*_c_ ≈ 0.5 for both samples. The curves were obtained by applying rectangular current pulses with a pulse width of 20 μs and a waiting time of 2 s.

**Table 1:** Values of some characteristic parameters of the NbRe/Au, NbRe/Py, and NbRe [19,22] microstrips. The thickness (in nm) of each layer is specified in parentheses. *L* is the distance between the voltage contacts, and *w* is the width of the microstrips. *T*_c_ is the critical temperature at 50% of the transition, ρ_10K_ is the resistivity in the normal state at *T* = 10 K, λ(0) and ξ(0) are, respectively, the London penetration length and the coherence length at zero temperature, and *D* is the quasiparticles diffusion coefficient.

Parameters	NbRe(20)/Au(5)	NbRe(15)/Py(4)	NbRe(60) [19]	NbRe(15) [22]

*L* (μm)	80.0 ± 0.1	80.0 ± 0.1	1000	100
*w* (μm)	10.0 ± 0.1	6.3 ± 0.1	100	10
*T*_c_ (K)	6.8 ± 0.1	5.6 ± 0.1	7.3	6.7
ρ_10K_ (μΩ·cm)	52 ± 5	124 ± 12	148	143
λ(0) (nm)	288 ± 14	494 ± 24	472	483
ξ(0) (nm)	6.3 ± 0.3	6.2 ± 0.3	4.80	—
*D* (×10^−4^ m^2^/s)	0.90 ± 0.06	0.70 ± 0.06	0.56	0.5

## Results and Discussion

### Superconducting characterization

**Figure 1:**
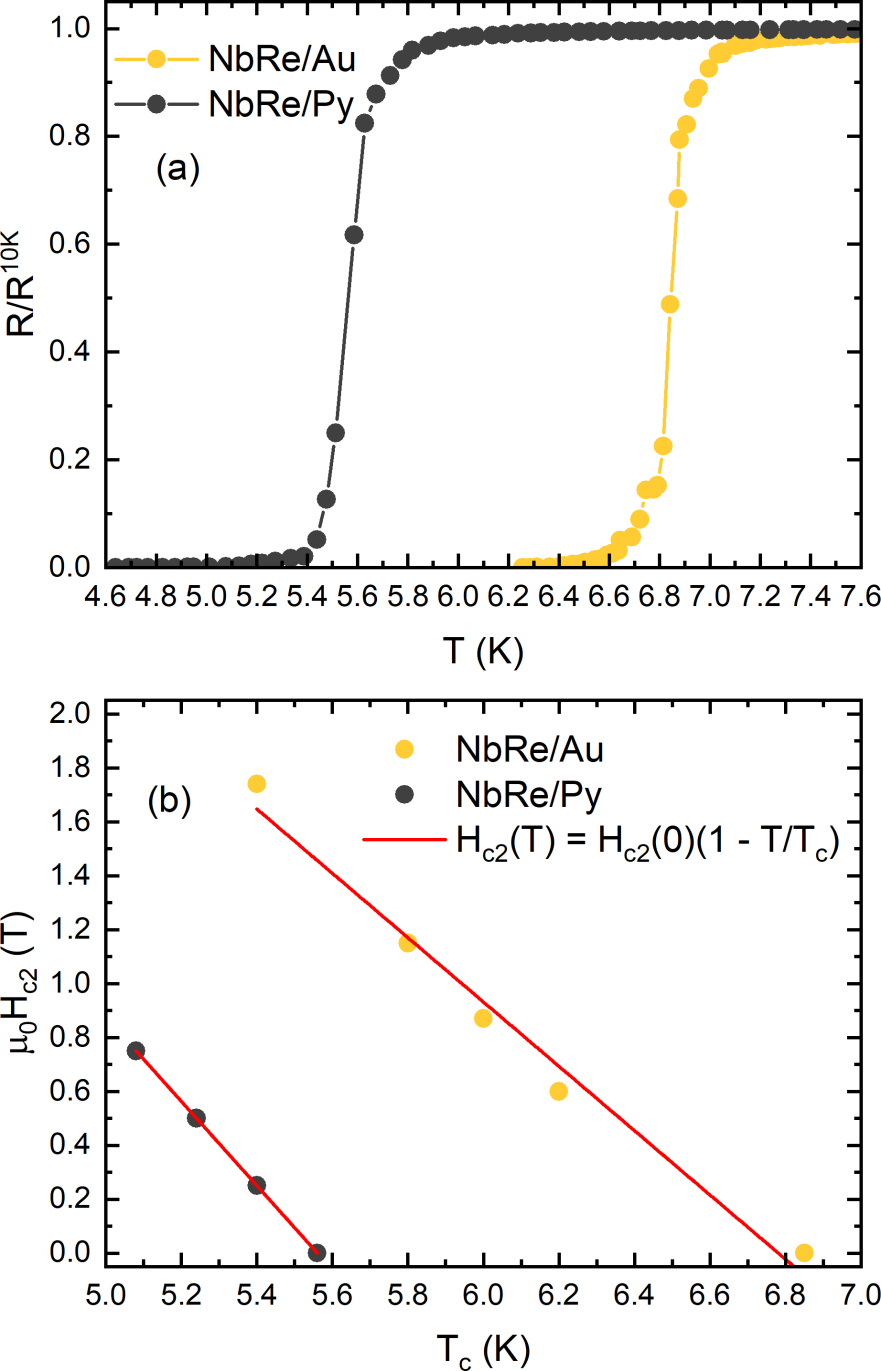
(a) Normalized resistive transitions and (b) μ_0_*H*_c2_(*T*) for the NbRe/Au and NbRe/Py bilayers. The red lines represent the linear fit to the data.

The normalized resistive transitions, *R*/*R*^10K^, of the two microbridges are reported in Figure 1a. The critical temperatures are 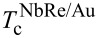 = 6.8 K and 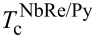 = 5.6 K. The obtained values are smaller than those reported for NbRe thin films of similar thickness [19,22], with a stronger reduction observed in the NbRe/Py bilayer, where the superconductivity is more suppressed by the proximity to the F layer. Notably, the magnitude of the *T*_c_ suppression observed in the NbRe/Py bilayer is comparable to that reported for NbRe/CuNi hybrid structures (
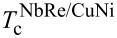
 = 5.86 K) reported in [22], supporting the conclusion that proximity to a ferromagnetic layer leads to a stronger suppression of superconductivity than in S/N bilayers. From *R*^10K^, the low-temperature resistivities of the two strips have been calculated, yielding 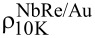 = 52 μΩ·cm and 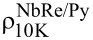 = 124 μΩ·cm. The significant difference in resistivity observed between the two bilayers cannot be ascribed to the different thicknesses of the NbRe layers since NbRe films of the same thickness exhibit nearly identical superconducting critical temperatures (around 7 K, Δ*T*_c_ ≈ 0.1 K) and low-temperature resistivities, of the order of ρ = 140 μΩ·cm [19]. In contrast, the capping layers are characterized by significantly different electrical resistivities, with Au being an excellent electrical conductor even at small thicknesses and Py films showing high resistivity in the ultrathin film regime [40–41]. To calculate the London penetration depth at *T* = 0 K, the formula λ(0) = 1.05 × 10^−3^
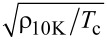
 [42] was used, resulting in λ^NbRe/Au^(0) = 288 nm and λ^NbRe/Py^(0) = 494 nm. The upper critical field, μ_0_*H*_c2_, as a function of temperature was obtained from *R*(*H*) measurements, and the corresponding experimental data are presented in Figure 1b. Assuming for μ_0_*H*_c2_(*T*) a linear dependence, it follows that the upper critical fields at *T* = 0 K of the two microbridges are 
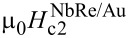
(0) = 8.30 T and 
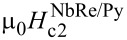
(0) = 8.70 T. From these values, the coherence length, ξ, at *T* = 0 K can be determined using the formula ξ(0) = [Φ_0_/2πμ_0_*H*_c2_(0)]^1^*^/^*^2^ yielding ξ^NbRe/Au^(0) = 6.3 nm and ξ^NbRe/Py^(0) = 6.2 nm. The quasiparticle diffusion coefficient *D* is evaluated from the slope of the μ_0_*H*_c2_(T) curve, since *D* = 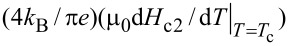. The quasiparticle diffusion coefficients are *D*^NbRe/Au^ = 0.9 × 10^−4^ m^2^/s and *D*^NbRe/Py^ = 0.7 × 10^−4^ m^2^/s. Table 1 summarizes the superconducting characteristics of the two microstrips. It is worth noting that the formulas employed were derived for single-layer superconducting films, whereas, in the present study, the microbridges are bilayer structures. As a result, the calculated parameters do not fully account for proximity-induced modifications of the electronic properties.

### Current–voltage characteristics and critical currents

**Figure 2:**
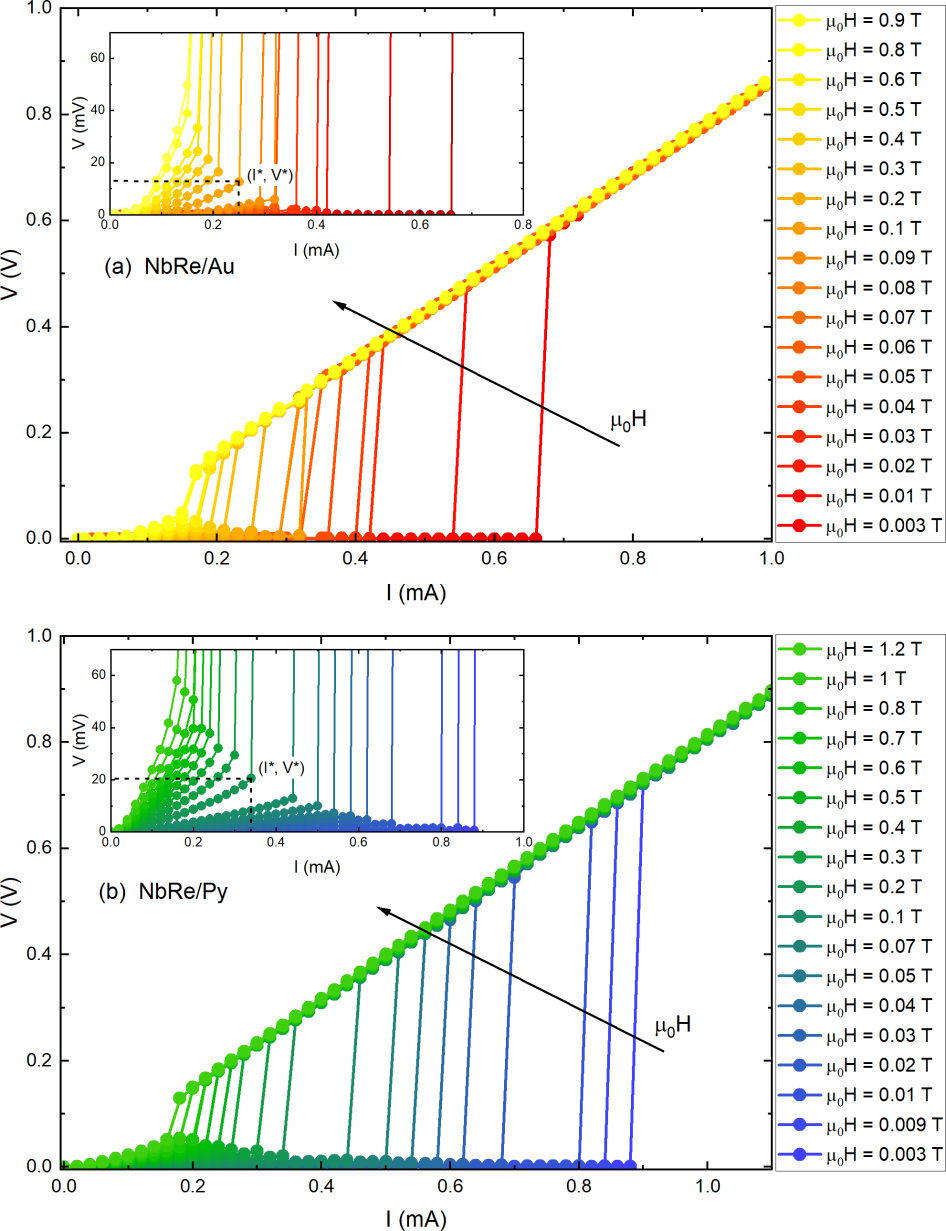
*I*–*V* curves of (a) NbRe/Au and (b) NbRe/Py bilayers at *t* ≈ 0.5 in different magnetic fields from 3 mT to the respective magnetic saturation field, *H*_SAT_. Insets: enlarged plot in the low-voltage region to show the nonlinear conductivity regime. The coordinates I* and V* denote the values of the instability current and voltage, respectively.

Figure 2 shows the *I*–*V* characteristics measured for the NbRe/Au and NbRe/Py microbridges at the reduced temperature *t* = *T*/*T*_c_ ≈ 0.5 as a function of the external magnetic field. The curves exhibit a zero-voltage regime at small bias currents where vortices remain pinned. It is clearly shown in the insets of Figure 2 that, as the current further increases, a nonlinear conductivity regime appears until I* is reached. When the magnetic field increases, the critical voltage at which the jump occurs also increases, while the values of I* decrease. The *I*–*V* curves allow one to determine the so-called saturation magnetic field, *H*_SAT_, that is, the field beyond which the jumps disappear and the transition to the normal state occurs smoothly. The values are 
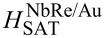
 = 0.9 T and 
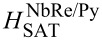
 = 1.2 T.

**Figure 3:**
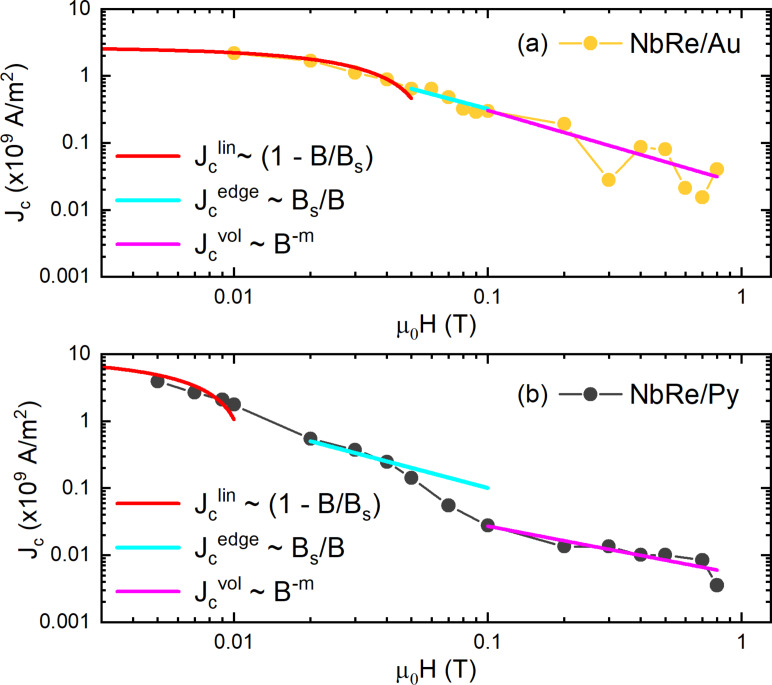
Magnetic field dependence for the critical current density at *t* ≈ 0.5 of NbRe/Au (a) and NbRe/Py (b). The red curves represent the dependence 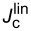 ∼ (1 − *B*/*B*_s_), the cyan curves the dependence 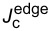
(*B*) ∼ *B*_s_/*B*, and the magenta curves the dependence 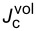 ∼ *B*^−^*^m^*.

To gain insight into the pinning mechanism at play, the behavior of the critical current density *J*_c_ = *I*_c_/*wd* as a function of the external magnetic field was analyzed as reported in [39,43]. The critical current values were measured based on a voltage criterion *V*_c_ = 20 μV, corresponding to *E*_c_ = *V*_c_/*L* = 0.25 V/m. Figure 3a and Figure 3b show the *J*_c_(*H*) curves on a double-logarithmic scale for NbRe/Au and NbRe/Py bilayers at *t* ≈ 0.5, respectively. For sufficiently low values of the magnetic field, the experimental data follow the linear dependence 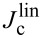
(*B*) = *J*_c_(*B* = 0)(1 − *B*/*B*_s_) (see red lines). The magnetic field *B*_s_ determines the point at which vortices overcome the edge barrier and enter the material, and it is expressed by the equation *B*_s_ = 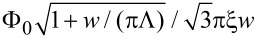 [43], where Λ = 2λ^2^/*d* is the Pearl penetration length. The theoretical values of *B*_s_ are 
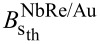
 = 5.3 mT and 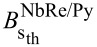 = 7.4 mT. The linear fits to the data were carried out fixing the value of *J*_c_ at μ_0_*H* = 0 T to the experimental values 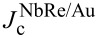
(0) = 2.64 × 10^9^ A/m^2^ and 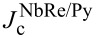
(0) = 8.7 × 10^9^ A/m^2^. The fits yield *B*_s_ equal to 
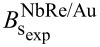
 ≈ 60 mT and 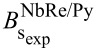 ≈ 11 mT for the two microbridges, respectively. Given the physical meaning of *B*_s_, the discrepancy between the experimental and theoretical values indicates that vortices overcome the edge barrier and penetrate the material only at magnetic fields larger than theoretically predicted. This enhancement of *B*_s_ can be understood by considering that the theoretical expression accounts for the suppression of the edge barrier, whereas the experimental *B*_s_ extracted from *J*_c_(*B*) reflects the onset of vortex penetration into the bulk, which, in the presence of bulk pinning, may require magnetic fields larger than the barrier-suppression one [44–45]. Moreover, the theoretical estimate of *B*_s_ relies on parameters such as Λ and ξ, evaluated using expressions derived for single-layer superconducting films. Since our samples are bilayer heterostructures, proximity-induced modifications of the superconducting properties are not fully taken into account, introducing an additional uncertainty in the theoretical value of *B*_s_. Considering edge quality, according to Plourde et al. [46], imperfections or geometric irregularities along the edges of the strip lower the effective entry field for vortices. The larger experimental *B*_s_ measured for the NbRe/Au microbridge thus points to a more efficient edge barrier with respect to the case of NbRe/Py bilayer where the value of *B*_s_ is smaller. The latter also exhibits a higher *J*_c_ compared to NbRe/Au, indicating a stronger vortex pinning force in this range. Furthermore, the results of NbRe/Py show a steeper slope than NbRe/Au. This could be related to a larger suppression and spatial inhomogeneity of the superconducting order parameter induced by the Py layer, which further decreases the value of the critical current density at low fields [34]. For magnetic fields between the experimentally obtained values of *B*_s_ and 0.1 T, *J*_c_(*B*) is well described by the dependence 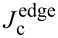
(*B*) ∝ *B*_s_/(4*B*), indicating the dominant role of the edge barrier pinning for vortex entry into the samples in this regime [43] (see cyan lines). Last, at higher magnetic fields, a gradual transition toward the dependence *J*_c_(*B*) ∼ *B*^−0.5^ is expected due to the onset of intrinsic volume pinning. For this reason, the experimental data were fitted to the equation 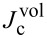
(*B*) = *A*_0_*B^−m^* leaving *m* as a free parameter (see magenta lines). For the two samples, the fit yields *m*_NbRe/Au_ = 1.12 and *m*_NbRe/Py_ = 0.75, indicating a stronger attenuation of the critical current density at high fields than expected. This suggests a weaker volume pinning effect compared to the theoretical result for both microbridges. These results demonstrate that edge-barrier pinning dominates over volume pinning in both samples, despite their large strip widths.

### Thermal effects

**Figure 4:**
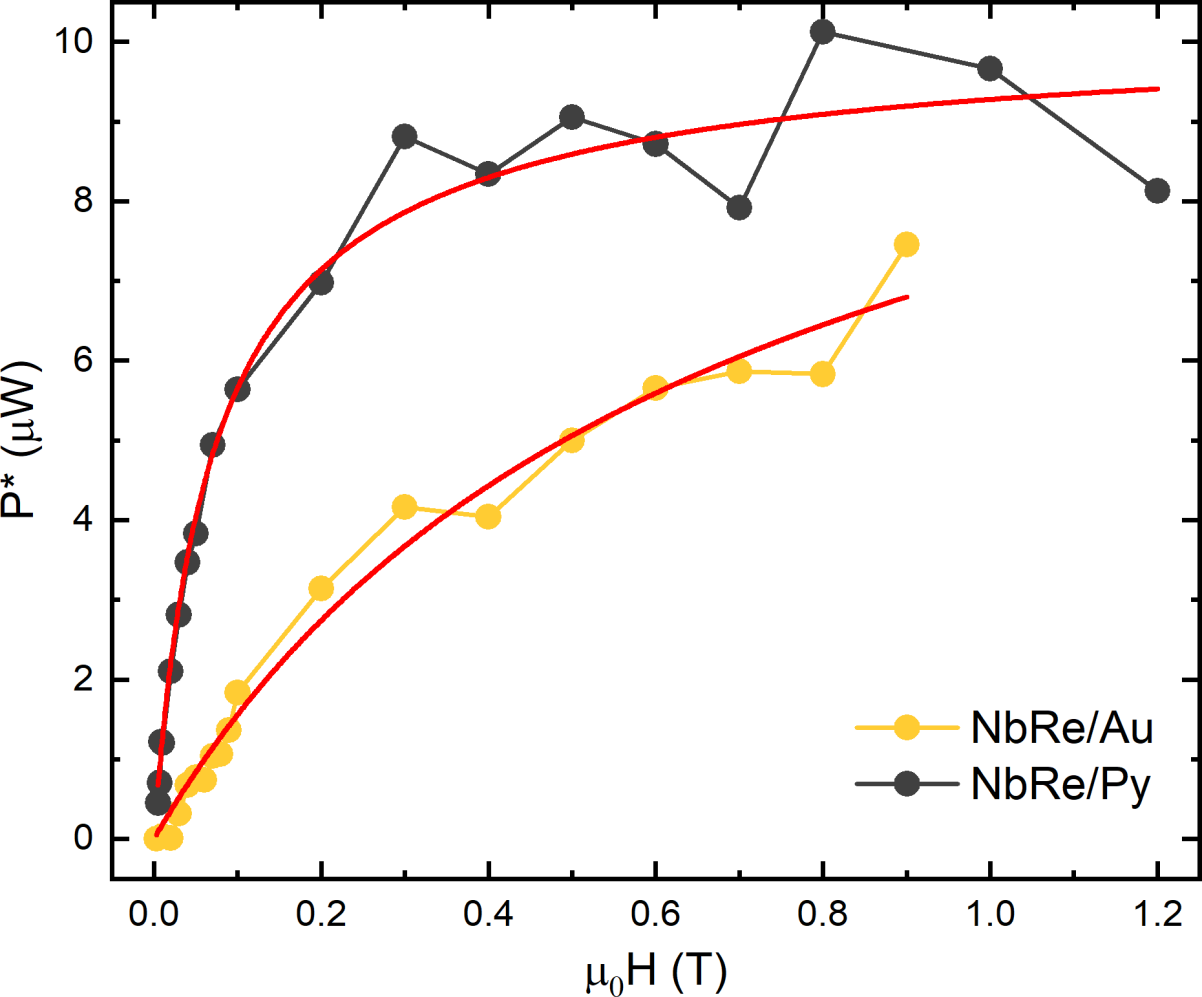
Dissipated power at the instability point as a function of the magnetic field at *t* ≈ 0.5 for NbRe/Au and NbRe/Py bilayers. The red solid lines are the fit to the data according to the BS theory [6].

Before starting the analysis of flux-flow instability at the critical velocity, it is crucial to rule out the possibility that the sudden transition to the normal state is due to Joule heating. To do this, according to Bezuglyj and Shklovskij (BS) theory [6], we analyzed the behavior of the critical power P* = I*V* as a function of the external magnetic field via the equation P* = *P*_0_ (1 − *a*), where *a* = 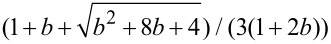 with *b* = *H*/*H*_T_. In the BS theory, *B*_T_ = μ_0_*H*_T_ is a thermal field and represents the magnetic field value beyond which thermal effects become dominant in the vortex instability dynamics. Figure 4 shows the behavior of the critical power as a function of μ_0_*H* for the two microbridges. In both cases, the fits (red solid lines) provide a good approximation of the experimental data. For NbRe/Au, the fit yields a thermal field 
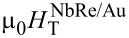
 = 1 T, which is larger than the corresponding saturation magnetic field values μ_0_*H*_SAT_ = 0.9 T. This ensures that, for the NbRe/Au bilayer, thermal effects do not play a significant role in the flux-flow instability phenomenon. In contrast, for the NbRe/Py bilayer, the thermal field is 
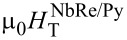
 = 0.1 T, which is much smaller than its saturation field μ_0_*H*_SAT_ = 1.2 T. This indicates that, for magnetic fields larger than 0.1 T, the NbRe/Py microbridge is affected by thermal effects and that the vortex instability dynamics is influenced by thermal dissipation mechanisms. The different impact of thermal effects observed in the two microbridges can be traced back to material-dependent and intrinsic geometrical factors. The thermal dissipation affecting the flux-flow instability is dominated by processes occurring within the superconducting microstrip itself, namely vortex motion and flux-flow dissipation, which are strongly influenced by the nature of the capping layer. In addition, the reduced strip width of the NbRe/Py microbridge leads to a less efficient heat evacuation toward the substrate, thereby enhancing the role of Joule heating and amplifying the impact of thermal dissipation on vortex dynamics. For this reason, and since, according to the LO theory, τ_E_ can be reliably estimated only when *J*_c_(*H*) reflects a dominant edge-barrier pinning mechanism (i.e., in this case, in the interval [*Bs*; 0.1 T] for both bridges), the critical vortex velocity and the corresponding quasiparticle relaxation time will be reported and compared at μ_0_*H* = 0.1 T.

### Vortex critical velocity and quasiparticles relaxation time

**Figure 5:**
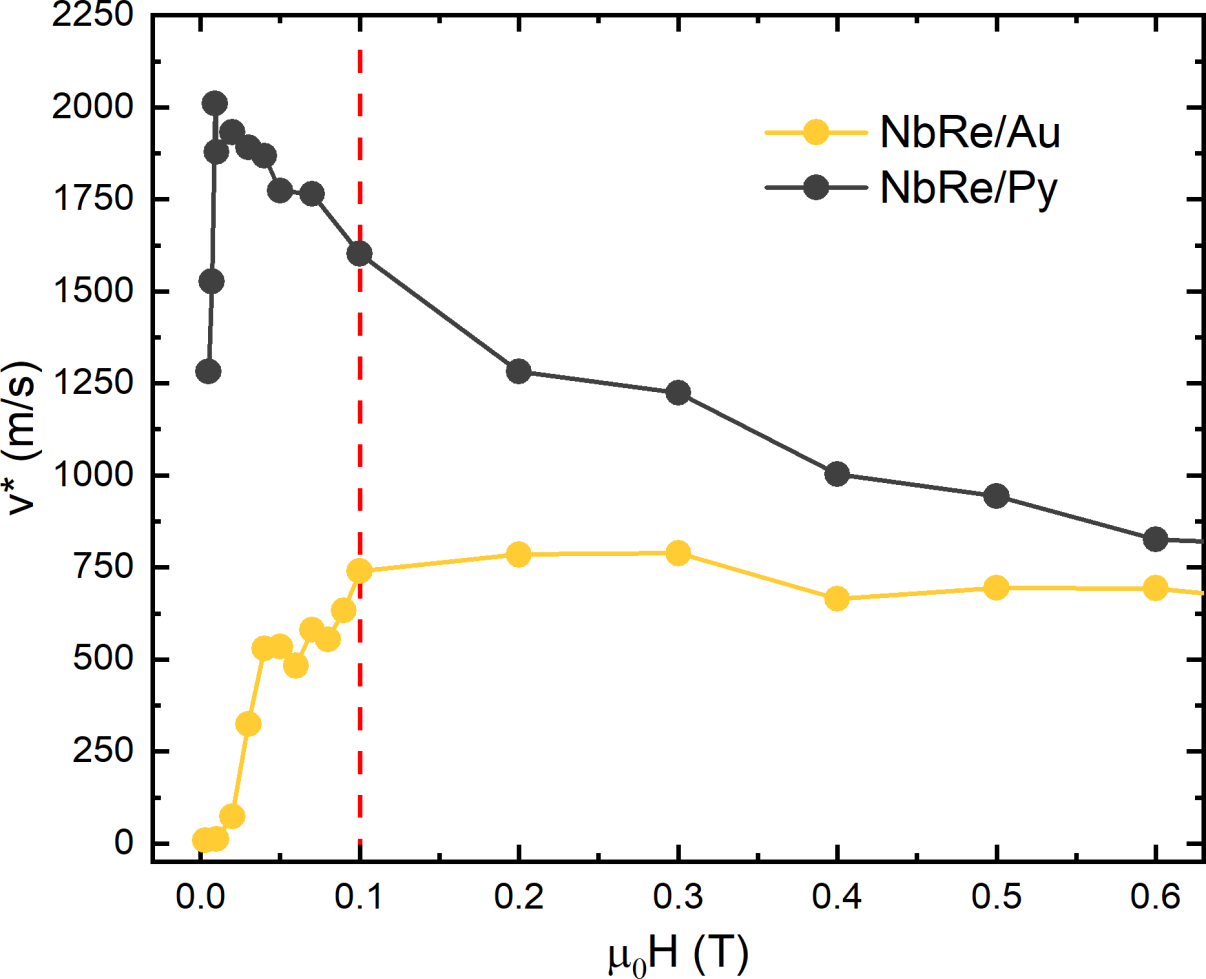
Vortex critical velocity as a function of the magnetic field for NbRe/Au and NbRe/Py at *t* ≈ 0.5. The red dashed line indicates the thermal field of NbRe/Py.

The dependence of v* as a function of the magnetic field is reported for both the NbRe/Au and the NbRe/Py bridges at *t* ≈ 0.5 in Figure 5. The figure clearly shows that the critical velocity values measured for the NbRe/Py are larger than those obtained for the NbRe/Au microstrip. The critical velocity increases with *H* up to approximately 0.1 T for the NbRe/Au sample and up to 0.01 T for NbRe/Py. This behavior can be linked to the fact that, in this regime, fluxons do not penetrate the film with a uniform front but advance with an irregular front characterized by protrusions. Thus, the magnetic field is non-uniformly distributed in the film, and preferential stationary movement channels for the vortices are generated [5,47]. At larger fields, the NbRe/Au sample displays a nearly field-independent v*, as predicted by Larkin–Ovchinnikov theory. In contrast, the NbRe/Py sample shows a decreasing v* with increasing *H*, consistent with previous experimental observations [48–50]. Furthermore, at larger fields, because of the small *H*_T_, the critical velocity in the NbRe/Py sample is limited by Joule dissipation. The latter facilitates the breakdown of superconductivity and reduces the vortex velocity in the bridge. Despite that, the S/F bilayer exhibits a significantly higher v* than the S/N bilayer. This enhancement may be attributed to the enhanced quasiparticle scattering and increased disorder introduced by the Py layer. At the field of 0.1 T, the microbridges reach critical vortex velocities values of v*^NbRe/Au^ ≈ 740 m/s and v*^NbRe/Py^ ≈ 1600 m/s, corresponding to a relaxation time of 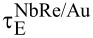 ≈ 150 ps and 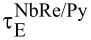 ≈ 24 ps.

The values of τ_E_ obtained for the heterostructures can be compared to those reported in the literature for single NbRe microstrips of similar geometric dimensions (*d*^NbRe^ = 15 nm, *L*^NbRe^ = 100 μm, and *w*^NbRe^ = 10 μm) [22]. In this case, a relaxation time equal to 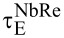 ≈ 200 ps at *t* ≈ 0.5 was reported. As a result, while the presence of the Au overlayer does not produce a significant reduction in the relaxation time, the ferromagnetic Py layer leads to a much more pronounced decrease of τ_E_, by almost one order of magnitude. The result observed in the NbRe/Au hybrid can be understood by estimating 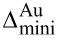 from Equation 3. Being 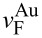 = 1.4 × 10^6^ m/s [36], and assuming that the mean free path is limited by the thickness *d*_Au_, the electron diffusion coefficient is *D*_Au_ = 2.3 × 10^−3^ m^2^/s. This value yields a coherence length of ξ_Au_ ≈ 30 nm at the measurement temperature (*T* = 3.5 K). Because ξ_Au_ ≫ *d*_Au_, the layer is completely proximized (ξ_N_ = *d*_Au_), and 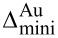 ≈ Δ^NbRe^. Therefore, the escape of quasiparticles from the vortex core is not effectively facilitated by the Au layer. As a consequence, the relaxation time does not exhibit any significant reduction and remains mainly governed by the underlying NbRe layer. The small improvement in the energy relaxation time could be related to the presence of the interface that moderately influences the quasiparticle relaxation mechanisms of the system [26]. In contrast, in an S/F system with a strong ferromagnet such as Py, the superconducting order parameter is strongly depressed over a very short distance (ξ_Py_ = 2 nm [33]) due to the large exchange field. Moreover, since Py is a disordered alloy, quasiparticles experience an enhanced scattering rate, further reducing the coherence of the induced correlations and promoting faster relaxation rates.

Finally, it is important to consider the possible role of thermal dissipation in the relaxation process. While a quantitative evaluation of the thermal effects is not straightforward, some considerations can be done. First, in both samples, the NbRe layer is in contact with the substrate. Therefore, the dominant heat dissipation channel into the thermal bath is common to both systems and does not affect the relative comparison between Au- and Py-capped samples. Consequently, substrate-related thermal effects can be considered equivalent, and the observed differences can be attributed to the specific properties of the capping layers and their interaction with the superconducting film. In this respect, Au and Py are expected to affect the vortex dynamics in qualitatively different ways when considering thermal dissipation and phonon-mediated quasiparticle recombination. In fact, Au is a non-magnetic metal with high electronic and thermal conductivity, providing a more effective heat removal. This is confirmed from the analysis performed in the framework of Bezuglyj and Shklovskij theory [6], from which it emerged that the NbRe/Au bilayer is less affected by thermal effects at the instability current. In contrast, Py is a ferromagnetic and more disordered metal, characterized by enhanced scattering processes. The latter can, in principle, provide an efficient energy relaxation channel, promoting faster quasiparticle recombination. However, this effect competes with additional spin-related relaxation channels due to the splitting of the electronic band, which can lead to a slower recovery of the superconducting condensate. Therefore, in general, the net effect of a disordered ferromagnetic capping layer on the critical velocity is not trivial and depends on the balance between different mechanisms. Our experiment suggests that, for these specific systems, disorder-related mechanisms play a dominant role in setting the value of v* with respect to the thermal effects.

## Conclusion

NbRe-based heterostructures with different capping materials have been fabricated and electrically characterized by measuring their *I*–*V* characteristics under external magnetic fields. From the current and voltage values at the instability points, the behavior of the critical vortex velocity as a function of the magnetic field was investigated to understand how normal metals and ferromagnetic materials affect vortex motion. The experimental data show that the NbRe/Py bilayer exhibits larger critical vortex velocities than those of the NbRe/Au structure. Furthermore, the estimation of the energy relaxation time at 0.1 T yields 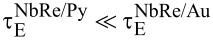. The comparison with the relaxation time reported for single NbRe microbridges indicates that, while the Au overlayer does not substantially modify the intrinsic relaxation dynamics of the superconducting layer, the presence of Py leads to a pronounced decrease in τ_E_ by almost one order of magnitude. This behaviour can be understood both in terms of proximity-induced modifications of the superconducting energy gap and the additional quasiparticle scattering present in the proximity coupled layers. In the NbRe/Au bilayer, the induced minigap in Au is comparable to that of NbRe, and therefore Au does not enhance the quasiparticle escape from the vortex cores. In contrast, in the NbRe/Py system, the more efficient quasiparticle energy dissipation is due to both the strong depression of the superconductivity and an increased scattering rate in the disordered Py layer. These findings confirm that spectral proximity effects play a key role in determining the vortex dynamics in hybrid systems.

## Data Availability

Data generated and analyzed during this study is available from the corresponding author upon reasonable request.
